# Characterization of the complete chloroplast genome of Yuanjiang wild Ichang papeda (*Citrus cavaleriei*) in China

**DOI:** 10.1080/23802359.2020.1820397

**Published:** 2020-09-16

**Authors:** Zi-hao Zhang, Chun-rui Long, Yan Jiang, Shi-zao Yang, Jun Zhao, Shao-Hua Wang

**Affiliations:** a Institute of Tropical and Subtropical Cash Crops, Yunnan Academy of Agricultural Sciences, Baoshan, P. R. China; b College of Horticulture, Fujian Agriculture and Forestry University, Fuzhou, P. R. China; c Institute of Dehong Tropical Agriculture Research Institute of Yunnan, Ruili, P. R. China

**Keywords:** *Citrus cavaleriei*, wild Ichang papeda, chloroplast genome, phylogenetic analysis

## Abstract

Ichang papeda (*Citrus cavaleriei*) is an endemic perennial plant in China. In this study, we assembled and annotated the complete chloroplast genome of Yuanjiang wild Ichang papeda using Illumina Hiseq-2500 sequencing data. The chloroplast genome is constituted of 160,996 bp, containing a 87,634 bp large single-copy region, a 18,762 bp small single-copy region, and a pair of 27,300 bp inverted repeat regions. The chloroplast genome contains 114 unique genes, including 80 protein-coding genes, 30 tRNAs and 4 rRNAs. Phylogenetic analysis showed that the relationship between the chloroplast gennomes of *C. cavaleriei* and *C*. *reticulata* is the closest, which consistently support their chloroplast relationships.

Ichang papeda (*Citrus cavaleriei*, once named as *Citrus ichangensis*), an original *Citrus* species with large wing leaves, is firstly discovered in Xingshan county, Hubei province of China (Chen et al. 2012; Yang et al. 2017). Nowadays, its distributions in Yunnan, Hunan, Guangxi, Guizhou, Sichuan, Chongqing and Shanxi provinces of China have also been reported. The Yunnan province of China, an important *Citrus* original center, is rich of endemic and wild *Citrus* species (Wu et al. 2018). And the wild Ichang papeda population in Yuanjiang county of Yunnan province is recognized the largest and the oldest (Chen et al. 2012). Current researches on Yuanjiang wild Ichang papeda were mainly focused on its genetic diversity and evolution from aspects of phenotype, stress resistance, molecular markers and so on (Chen et al. 2012; Yang et al. 2017). In the present study, to provide genetic information of Yuanjiang wild Ichang papeda, we assembled and annotated the complete chloroplast genome, and investigated its phylogenetic relationships with other *Citrus* species.

The specimen of Yuanjiang wild Ichang papeda was isolated from Mountain Nizubai, Yicibei village, Wadie town, Yuanjiang county, Yunnan province, China (23°29′33.036″N; 102°17′9.276″E) and samples were deposited at the Institute of Tropical and Subtropical Cash Crops, Yunnan Academy of Agricultural Sciences. The leaf genomic DNA of Yuanjiang wild Ichang papeda was extracted using the CTAB method (Xie et al. 2020) and stored at the Institute of Tropical and Subtropical Cash Crops, Yunnan Academy of Agricultural Sciences (No. YJYCC01). The whole genomic DNA re-sequencing was performed on the Illumina Hiseq-2500 platform to generate 125 bp pair-end reads (BIG, Shenzhen, CA, CHN). Totally, we obtained about 7 G high quality reads, which were aligned to chloroplast genomes of six *Citrus* species according to Wu et al. (2019) and Xu et al. (2019). Chloroplast genome assembly and annotation were performed according to the method described by Xu et al. (2019), and the annotated chloroplast genome has been deposited in Genbank with the accession number MT880606.

The complete chloroplast genome of *C. cavaleriei* is 1,60,996 bp in size, containing a large single-copy region of 87,634 bp, a small single-copy region of 18,762 bp, and a pair of inverted repeat regions of 27,300 bp. Sequence annotation identified 114 unique genes from the *C*. *cavaleriei* chloroplast genome, including 80 protein-coding genes, 30 tRNA genes and 4 rRNA genes. Among the 114 unique genes, nine protein-coding genes (i.e. *ndhB*, *rpl2*, *rpl22*, *rpl23*, *rps7*, *rps12*, *rps19*, *ycf2* and *ycf15*), seven tRNA genes (i.e. *trnA-UGC*, *trnI-CAU, trnI-GAU*, *trnL-CAA*, *trnN-GUU*, *trnR-ACG* and *trnV-GAC*) and all the 4 rRNA genes (*rrn4.5*, *rrn5*, *rrn16* and *rrn23* rRNAs) occur in double copies, and others occur as a single copy. The overall nucleotide composition of the chloroplast genome is: 30.47% A, 31.07% T, 19.58% C, and 18.88% G, with the total GC content of 38.46%.

By using the complete chloroplast genomes of *C*. *cavaleriei*, 20 plant species from Rutaceae family and *Ailanthus altissima* (as outgroup), a maximum likelihood phylogenetic tree (Xu et al. 2019). Results showed that the relationship between the *C*. *cavaleriei* and *C*. *reticulata* chloroplasts is the closest (Figure 1), which consistently support their chloroplast relationship that has been put forward by many scientists (Nicolosi et al. 2000; Penjor et al. 2013; Yang et al. 2017).

**Figure 1.**
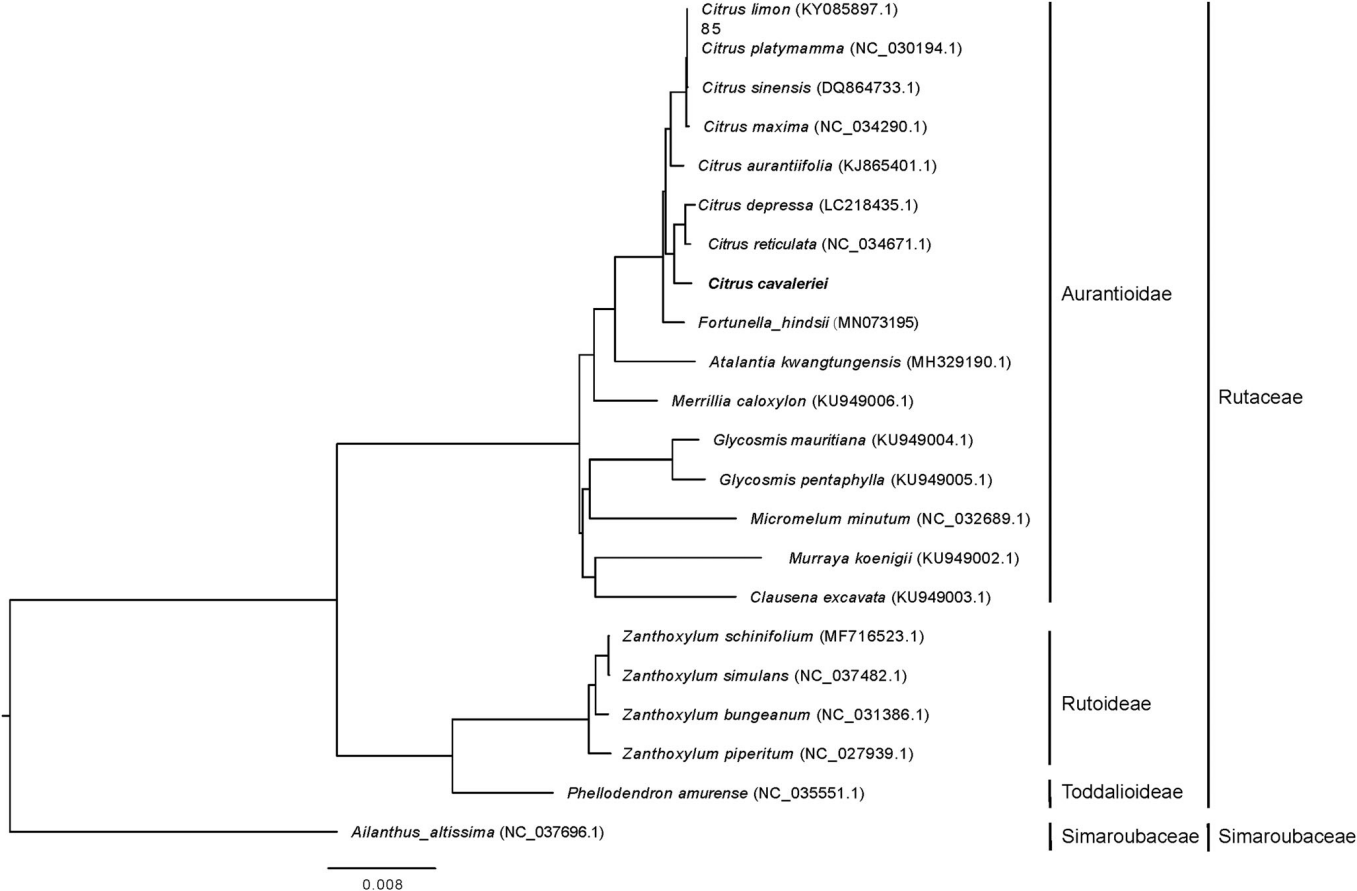
The maximum-likelihood phylogenetic tree constructed using the complete chloroplast genome sequences of *Citrus cavaleriei* and 20 plant species from the Rutaceae with *Ailanthus altissima* as outgroup. The phylogenetic tree was constructed according to the method and result described by Xu et al. (2019).

## Data Availability

The raw data that support the findings of this study are openly available in NCBI Sequence Read Archive at [http://www.ncbi.nlm.nih.gov/bioproject/658640] under the BioProject ID PRJNA658640 and the annotated chloroplast genome has been deposited in Genbank [https://www.ncbi.nlm.nih.gov/genbank/] under the reference number MT880606.
